# The correlation between cutout and eccentric distance (ED) of the cephalic fixator tip in geriatric intertrochanteric fractures with internal fixation

**DOI:** 10.1186/s13018-022-03153-x

**Published:** 2022-05-13

**Authors:** Yun-fa Yang, Jian-wen Huang, Xiao-sheng Gao, Zhong-he Xu

**Affiliations:** Department of Orthopaedic Surgery, Guangzhou First People’s Hospital, The Second Affiliated Hospital, School of Medicine, South China University of Technology, 1 Panfu Road, Guangzhou, 510180 Guangdong China

**Keywords:** Eccentric distance, Intertrochanteric fractures, Internal fixation, Cutout, Artificial intelligence

## Abstract

**Background:**

The location of cephalic fixator tip with different eccentric distance (ED) should have different risks of cutout. This study aims to evaluate the cephalic fixator tip position by measuring ED of the cephalic fixator tip in geriatric ITF patients with single-screw cephalomedullary nail (SCMN) fixation and analyze the correlation between the cutout and the ED.

**Methods:**

Firstly, we assumed all the femoral head was a regular sphere and standardized the radius of the femoral head (*R*_FD_) as “3” no matter how big the *R*_FD_ was for complete match of the Cleveland zone system and convenient identification of artificial intelligence. Secondly, we measured the ED of the cephalic fixator tip by calculating the distances from the cephalic fixator tip to the geometric central axis of the femoral neck and head on both AP view and lateral view radiographs. Thirdly, we evaluated all the ED of the cephalic fixator tip in the eligible 123 geriatric ITF patients and analyzed the correlation between the cutout and the ED.

**Results:**

The ED in cutout group (1.25 ± 0.43) is much bigger than that in non-cutout group (0.64 ± 0.34) with significant difference (OR = 50.01, 95% CI 8.42–297.19, *p* < 0.001). The probability of cutout increased with ED increasing, especially when “ED ≥ 1.” The best cutoff value of ED for predicting cutout was “1.022” (“1.022” was just a little bit more than 1/3 times of R_FD_ because “*R*_FD_ = 3,” sensitivity = 73.3%, specificity = 86.1%, and AUC = 0.867, *p* < 0.001).

**Conclusion:**

ED is suitable for evaluation of the cephalic fixator tip position for predicting cutout in geriatric ITF patients with SCMN fixation, and ED can potentially be used as artificial intelligence application during surgery. The smaller the ED, the lower the cutout rate. For avoiding cutout, the ED of the cephalic fixator tip should be less than one-third times of the radius of the femoral head.

## Background

The incidence of intertrochanteric fractures (ITF) is dramatically increased due to the aging population in all over the world. The social burden of ITF significantly ascends because geriatric ITF patients usually have some comorbidities, mortality, and aging-related complications. Actually, surgical treatment is almost the best choice for these geriatric ITF patients if they have no special contraindications for surgery. Nowadays, cephalomedullary nails (CMN) have been widely used for geriatric ITF patients’ internal fixation because of their inherently biomechanical advantages and good clinical outcomes. However, postoperative implant failure (such as cutout) rate ranged in 1.85–20.5%, which remains a great challenge to orthopedists globally [1–4].

Actually, cutout is highly associated with the location of the cephalic fixator tip in ITF patients with internal fixation [5–10]. And the Cleveland zone system is easily available for the surgeon to evaluate the cephalic fixator tip placement [2, 5–7, 11–16]. In Cleveland zone system, the femoral head is divided into superior, central, and inferior thirds on the anteroposterior radiograph and into anterior, central, and posterior thirds on the lateral radiograph, resulting in that the femoral head is divided into nine separate zones for evaluating the cephalic fixator tip position [5].

However, cutout still occurs in the patients with the cephalic fixator tip located in Cleveland Zone 5. The mechanical effect of the cephalic fixator tip in Cleveland Zone 5 is different because the eccentric distance (ED) in the marginal region of Cleveland Zone 5 is much bigger, and that is probably why cutout still occurs in the patients with the cephalic fixator tip placed in Cleveland Zone 5.

Therefore, we hypothesized that the placement of cephalic fixator tip in different ED should have different risks of cutout. We aimed to (1) measure the ED of cephalic fixator tip, and (2) analyze the relation between the cutout and the ED of cephalic fixator tip in the geriatric ITF patients with internal fixation.

## Methods

We measured the ED of cephalic fixator tip and analyzed the correlation between the cutout and the ED of cephalic fixator tip in the geriatric ITF patients with single-screw cephalomedullary nail (SCMN) fixation.

### ED measurement

We located the tip of cephalic fixator in the coordinate diagram (an *x–y* plot) of femoral head and evaluated the cephalic fixator position by individually measuring the ED of the cephalic fixator tip in ITF patients with SCMN fixation.

**Fig. 1:**
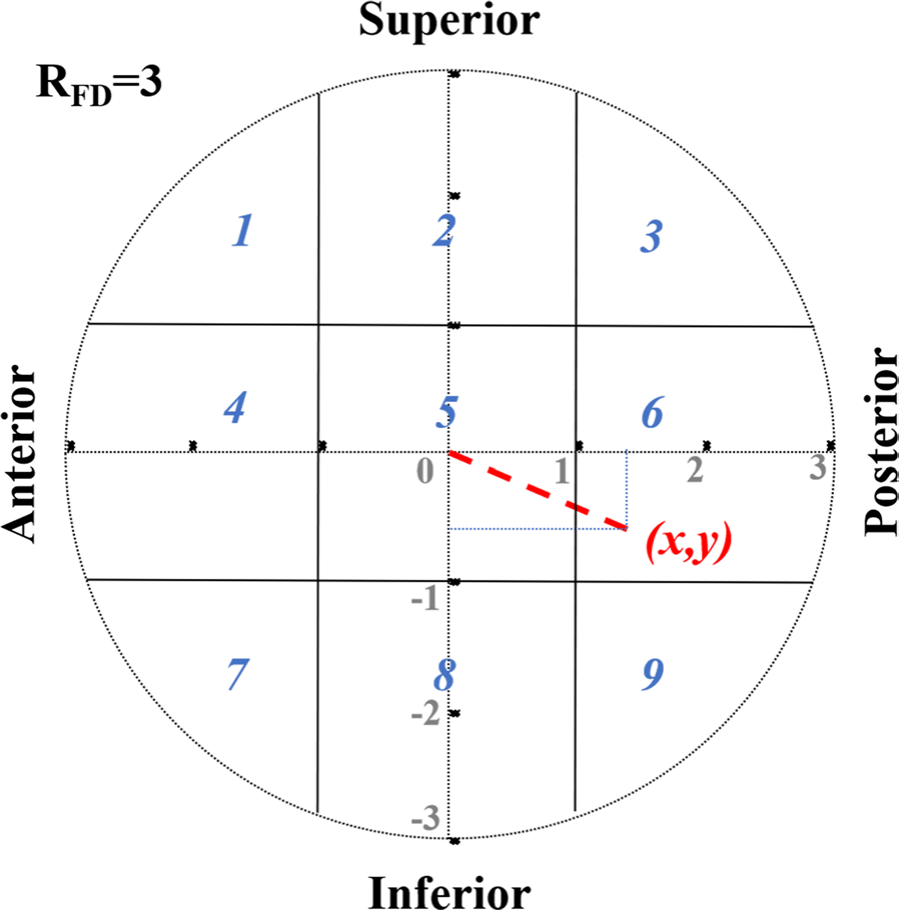
The coordinate graph (an *x–y* plot) for eccentric distance (ED) calculation of the cephalic fixator tip in the femoral head. No matter how big the radius of the femoral head (*R*_FD_) was, we assumed the *R*_FD_ as “3” (*R*_FD_ = “3,” without any unit) for good match of Cleveland zone system and easy calculation. ED of the cephalic fixator tip (*x*, *y*) is the distance from the circle center to the tip point of (*x*, *y*)

Firstly, we assumed all the femoral head was a regular sphere and standardized that all the radius of the femoral head (*R*_FD_) was “3” (*R*_FD_ = “3,” without any unit) no matter how big the *R*_FD_ was in order for complete match of the Cleveland zone system and convenient identification of the artificial intelligence (Fig. 1).

**Fig. 2:**
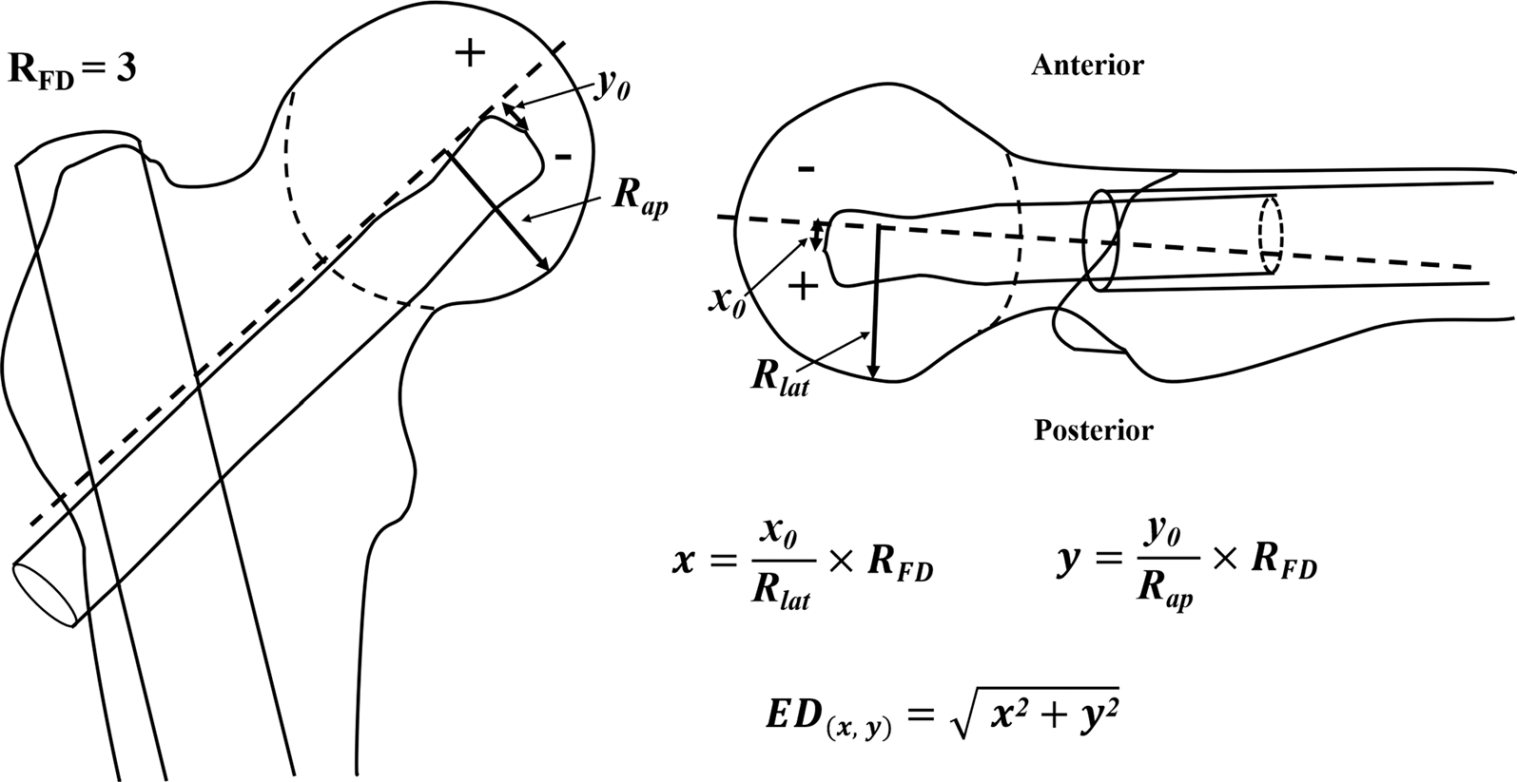
The measurement and calculation of ED. No matter how big the radius of the femoral head (*R*_FD_) was, we assumed the *R*_FD_ as “3” (*R*_FD_ = “3,” without any unit) for good match of the Cleveland zone system and easy calculation. “*x*_0_, *y*_0_, *R*_ap_, *R*_lat_” were actual measured values. The ED of the cephalic fixator was calculated by the distances from the cephalic fixator tip (*x*, *y*) to the geometric central axis in the femoral neck and head on both the AP view radiograph (*y* = *y*_0_/*R*_ap_ × *R*_FD_) and the lateral view radiograph (*x* = *x*_0_/*R*_lat_ × *R*_FD_), resulting in “ED_(*x*,*y*)_ = (*x*^2^ + *y*^2^)^1/2^.” The value of “*x*” or “*y*” was defined as positive if the cephalic fixator tip was on the superior or posterior, and as negative if the cephalic fixator tip was on the inferior or anterior when compared with the axis of femoral head

**Fig. 3:**
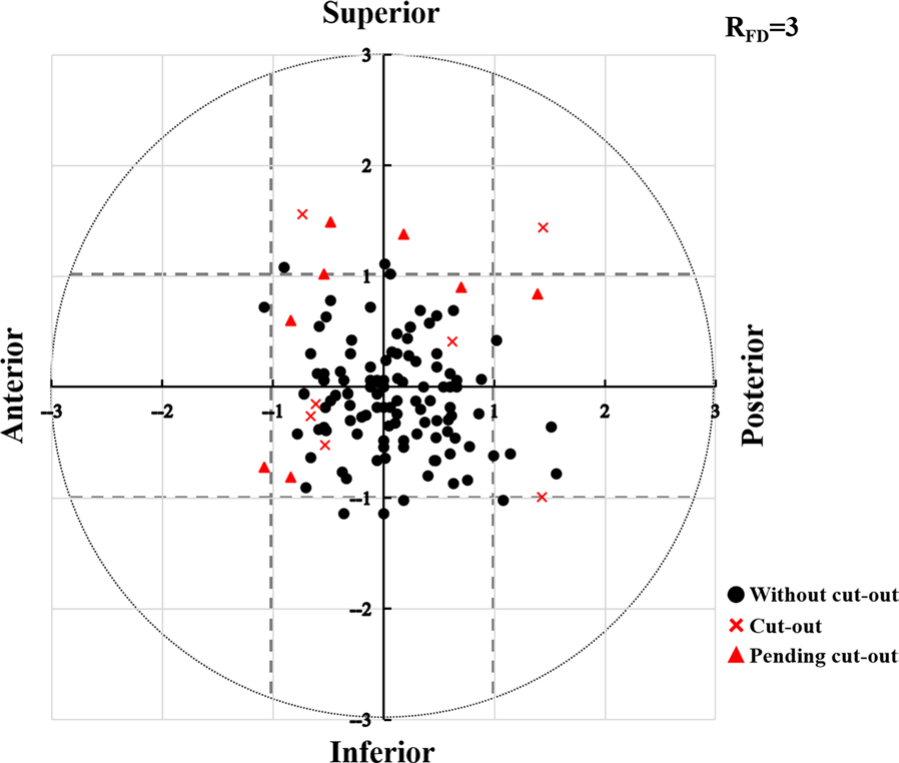
The coordinate graph (an *x–y* plot) of all the cephalic fixator tip placement in the femoral head of the eligible geriatric 123 ITF patients

Secondly, we measured the ED of the cephalic fixator tip by calculating the distances from the cephalic fixator tip to the geometric central axis in the femoral neck and head on both lateral view radiograph (*x* = *x*_0_/*R*_lat_ × *R*_FD_) and AP view radiograph (*y* = *y*_0_/*R*_ap_ × *R*_FD_), resulting in “ED_(*x*,*y*)_ = (*x*^2^ + *y*^2^)^1/2^” (Fig. 2). The femoral neck geometric central axis was a straight line through both the femoral head geometric center and the femoral neck geometric center [17]. “*x*_0_, *y*_0_, *R*_ap_, *R*_lat_” were actual measured values. The value of “*x*” or “*y*” was defined as positive if the cephalic fixator tip was on the superior or posterior, and as negative if the cephalic fixator tip was on the inferior or anterior referencing the axis of femoral head. Thus, we could intuitively locate the tip of cephalic fixator in the coordinate diagram of the femoral head and easily calculate the ED (Fig. 3). ED of the point (*x*, *y*) is the distance from the circle center to the point of (*x*, *y*) (Fig. 1).

### The correlation between the cutout and the ED

This observational study was approved by the Ethics Committee of our Hospital and conducted on patients with ITF treated surgically and followed in our hospital between September 2016 and August 2020, retrospectively. There were totally 187 ITF patients treated and followed-up in our hospital during this period.

The exclusion criteria: (1) age < 65 years, (2) pathological fractures, (3) loss of preoperative or postoperative radiographs, (4) internal fixation was dual-screw cephalomedullary nail or plate system, and (5) patients without any implant failures while radiological follow-up less than 6 months.

The eligible ITF patients with SCMN fixation were divided into the cutout group and non-cutout group according to whether cutout or not. We measured all the ED of the cephalic fixator tip in the eligible patients. Then, we analyzed the correlation between cutout and clinical data including the ED.

The clinical data including age, gender, fracture site, fractures classifications according to the AO Foundation and Orthopaedic Trauma Association system (AO/OTA), American Society of Anesthesiologists (ASA) classification, bone quality, anesthesia, fixation type, reduction quality, Cleveland zone system, and ED of cephalic fixator tip were analyzed.

All of the radiological parameters were evaluated by two observers (JWH and XSG). Fracture classification was determined on preoperative AP radiographs by the AO/OTA system (2018 version) [18]. Bone qualities were evaluated by the Singh index ratio on preoperative AP radiographs [19]. Reduction qualities were graded into three conditions (poor, acceptable, and good) based on the criteria developed by Baumgaertner [20].

The relation between the ED and the cutout was analyzed. The definition of cutout was the upper extrusion of the cephalic fixator from the femoral head. The pending cutout was the presence of over 20° decrease in neck-shaft angle (NSA) on the AP view while no cephalic fixator penetration in the last radiographic follow-up compared with the NSA at the first radiograph right after surgeries.

### Statistical analysis

The occurrence of cutout was defined as the dependent variable. Univariate analysis of continuous and categorical variables was performed with Student’s *t* test and chi-square test, respectively. The fitting curve was used for the correlation between the ED value and the probability of cutout. All analyses above were performed using SPSS (IBM SPSS Statistic for Windows, Version 25.0. Armonk, NY: IBM Corp). All tests were two-sided, and the statistical significance was defined as the *p* value below 0.05. The receiver operating characteristic (ROC) curves were performed to assess cutoff value and the reliability of the ED in predicting cutout with MedCalc® Statistical Software version 19.5.6 (MedCalc Software Ltd., Ostend, Belgium).

## Results

A total of 123 eligible geriatric ITF patients were included in this full analysis. The 123 patients include 43 males and 80 females with the age of 80.4 ± 8.4 years. The follow-up ranged from 6 to 48 months with an average of 11.8 months. Overall, 15 ITF patients were found with cutout or pending cutout (cutout group). The remaining 108 ITF patients were without cutout (non-cutout group) (Fig. 3).

In the univariate analysis (Table 1), no significant differences were found in age, gender, fracture site, fracture classification, Singh index, anesthesia, ASA classification, fixation type, and reduction quality. Cephalic fixator tip placements evaluated by the ED had significant differences for cutout (*p* < 0.001).

**Table 1:** Univariate analysis of collected data

Factor	Overall (*n* = 123)	Non-cutout group (*n* = 108)	Cutout group (*n* = 15)	*p* value^†^	OR (95% CI)
Age (mean ± SD)	80.4 ± 8.40	80.3 ± 8.43	81.1 ± 8.46	0.744*	1.01 (0.95–1.08)
Gender				0.255^†^	2.35 (0.63–8.84)
Male	43 (35.0)	40 (37.0)	3 (20.0)		
Female	80 (65.0)	68 (63.0)	12 (80.0)		
Fracture site				0.781^†^	1.27 (0.43–3.77)
Left	71 (57.7)	63 (58.3)	8 (53.3)		
Right	52 (42.3)	45 (41.7)	7 (46.7)		
AO/OTA classification				0.108^†^	NA
31A1	62 (50.4)	58 (53.7)	4 (26.7)		
31A2	56 (45.5)	46 (42.6)	10 (66.7)		
31A3	5 (4.1)	4 (3.7)	1 (6.6)		
Singh index				0.428^†^	1.56 (0.52–4.68)
≤ 3	61 (49.6)	53 (49.1)	9 (60.0)		
> 3	62 (50.4)	55 (50.9)	6 (40.0)		
Anesthesia				0.598^†^	1.96 (0.42–9.27)
Spinal	95 (77.2)	82 (75.9)	13 (86.7)		
General	28 (22.8)	26 (24.1)	2 (13.3)		
ASA				0.719^†^	NA
2	54 (43.9)	46 (42.6)	8 (53.3)		
3	66 (53.7)	59 (57.4)	7 (46.7)		
4	3 (2.4)	3 (2.8)	0 (0.0)		
Fixation type (%)				0.559^†^	1.39 (0.46–4.21)
Blade	41 (33.3)	35 (32.4)	6 (40.0)		
Screw	82 (66.7)	73 (67.6)	9 (60.0)		
Reduction quality				0.176^†^	NA
Good	54 (43.9)	50 (46.3)	4 (26.7)		
Acceptable	47 (38.2)	38 (35.2)	9 (60.0)		
Poor	22 (17.9)	20 (18.5)	2 (13.3)		
ED value	0.71 ± 0.40	0.64 ± 0.34	1.25 ± 0.43	< 0.001*	50.01 (8.42–297.19)

*AO/OTA* AO Foundation and Orthopaedic Trauma Association, *ASA* American Society of Anesthesiologists, *ED* eccentric distance, *OR* odds ratio, *CI* confidence interval, *N/A* not applicable

*Student’s *t* test for continuous variables

^†^Chi-square test for categorical variables

**Fig. 4:**
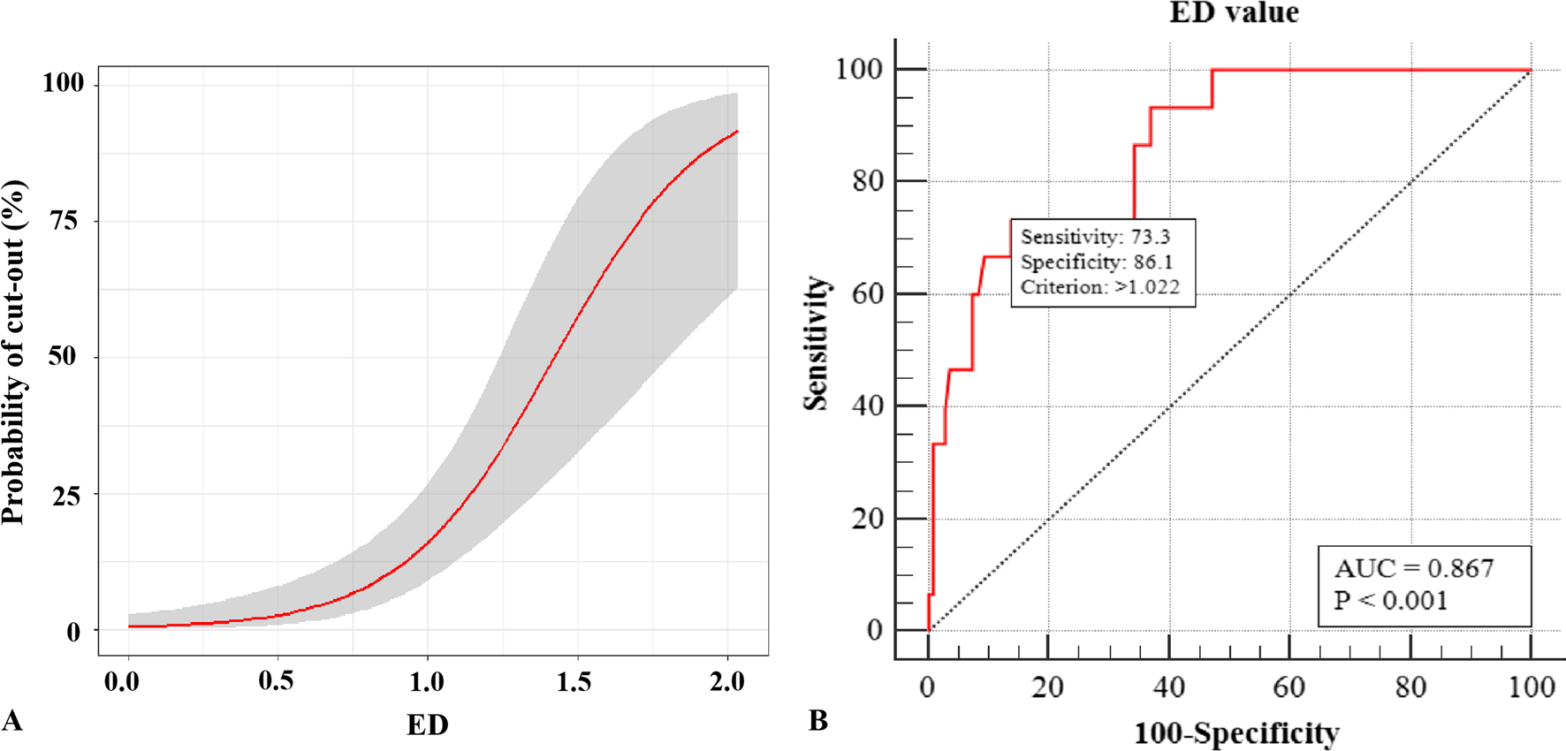
The graph **A** shows a fitting curve about predicted probability between cutout and ED. The probability of cutout increased with ED increasing (the gray filling area indicates a 95% CI of cutout rates in each ED) and the probability of cutout increased dramatically when “ED ≥ 1.” The graph **B** shows the ROC analysis between ED value and cutout rates. The best cutoff value of ED was 1.022 (just a little bit more than 1/3 times of *R*_FD_, sensitivity = 73.3%, specificity = 86.1%, AUC = 0.867, *p* < 0.001)

The predicted probability of cut-out correlated with ED was shown in the fitting curve of bivariate logistic regression (Fig. 4A), which indicated that the probability of cutout increased dramatically with the increase in ED, particularly when “ED ≥ 1.” The ROC analysis of ED is shown in Fig. 4B. The best cutoff value of ED was “1.022” with a sensitivity of 73.3% and specificity of 86.1% (area under the curve, AUC = 0.867, *p* < 0.001) (Fig. 4).

## Discussion

The occurrence of cutout in geriatric ITF with SCMN is highly associated with the implant placement, particularly the cephalic fixator tip within the femoral head. The center–center principle was the leading principle of the cephalic fixator tip position [2, 7, 12, 17, 21]. However, precise tools were still lacking on measuring the cephalic fixator tip position in the femoral head in the literature. We think that we can resolve the problem by measuring the ED of the cephalic fixator tip. In this study, we find that the ED measurement is a reliable evaluation tool of the cephalic fixator tip position in predicting cutout in the geriatric ITF patients with SCMN fixation. The occurrence of cutout rises with the increasing ED, and the rate of cutout increases dramatically once ED is over “1.022.” For avoiding cutout, the ED of the cephalic fixator tip should be less than one-third times of the radius of the femoral head.

### ED is significantly accurate for predicting cutout

The ROC curve demonstrates that the ED is significantly accurate for predicting cutout: The smaller the ED, the lower the cutout rate. The ED in cutout group (1.25 ± 0.43) is much bigger than that in non-cutout group (0.64 ± 0.34) with significant difference (OR = 50.01, 95% CI 8.42–297.19, *p* < 0.001). The probability of cutout increased with ED increasing, especially when “ED ≥ 1.” The best cutoff value of ED for predicting cutout was “1.022” (sensitivity = 73.3%, specificity = 86.1%, and AUC = 0.867, *p* < 0.001). Based on choosing fitting parameters of the cephalic fixators, placing cephalic fixator tip as centrally in femoral head as possible could decrease cutout even it was accompanied by the slightly superior or anterior placing. The smaller the ED, the lower the cutout rate.

### ED should be less than 1/3 times of the radius of the femoral head

The ROC curve demonstrates that the best cutoff value of ED of the cephalic fixator tip was “1.022” (with a sensitivity of 73.3%, specificity of 86.1%, and AUC of 0.867, *p* < 0.001). Actually, “1.022” was just a little bit more than 1/3 times of R_FD_ because “*R*_FD_ = 3” in this study. Our study discovered that the “slightly superior or anterior” can be determined quantitatively by the ED measurement which did not deviate one-third times of the radius of the femoral head as the center of the femoral head. Many studies had also demonstrated that central or inferior on AP view and central or posterior on lateral view within the femoral head were optimal options to prevent cutout [12, 17, 22–26]. In a word, ED of the cephalic fixator tip should be less than 1/3 times of the radius of the femoral head.

### ED may potentially be a good AI application during surgery

It is also easy to measure the ED because the numerical relationship of the ED is completely matched by the Cleveland zone system and we only calculate the ED on both the AP view and the lateral view radiographs. Besides, the ED is a relative number (the measurement with no complicated formula, regardless of magnification), which provides convenience in clinical usage. If we can set up relative software of ED measurement in C-arm X-ray machine, we may even use ED in artificial intelligence (AI) measurement in guiding the cephalic fixator placement just during surgeries.

### Limitations or weaknesses

However, there are still some limitations or weaknesses in this study. Firstly, the measurement of ED is based on the ideal condition that the femoral head is a regular sphere. However, the femoral head is not an exact regular sphere. Secondly, we only verify the applicability of ED in geriatric ITF patients with SCMN fixation; as a result, the conclusion may not be suitable for other internal fixations. Thirdly, we have no consideration on the depth of cephalic fixator tip. Actually, the depth of cephalic fixator tip usually measured by tip–apex distance (TAD) is so important in avoiding cutout. Thus, further studies are necessary to verify the clinical significance and application of ED.

## Conclusions

ED is suitable for evaluation of the cephalic fixator tip position for predicting cutout in geriatric ITF patients with SCMN fixations, and ED can potentially be used as artificial intelligent application during surgery. The probability of cutout increases with the ED increasing. For avoiding cutout, the ED of the cephalic fixator tip should be less than one-third times of the radius of the femoral head.

## Data Availability

The dataset supporting the conclusions of this study is available upon request by contacting the corresponding author, but the primary data were not shared because other studies related to these primary data were underway confidentially.

## Code availability

Software applications used in this study are listed as follows: IBM SPSS Statistic for Windows, Version 25.0. Armonk, NY: IBM Corp; Digimizer 5.4.4© 2005–2021 MedCalc Software Ltd.; MedCalc® Statistical Software version 19.5.6 (MedCalc Software Ltd., Ostend, Belgium; https://www.medcalc.org; 2020).
